# Tissue Distribution and Receptor Activation by Somapacitan, a Long Acting Growth Hormone Derivative

**DOI:** 10.3390/ijms21041181

**Published:** 2020-02-11

**Authors:** Maj Petersen, Prafull S. Gandhi, Jens Buchardt, Tomas Alanentalo, Johannes Josef Fels, Nils Langeland Johansen, Peter Helding-Kvist, Knud Vad, Peter Thygesen

**Affiliations:** 1 Global Drug Discovery, Novo Nordisk A/S, 2760 Måløv, Denmark; 2 Global Research Technologies, Novo Nordisk A/S, 2760 Måløv, Denmark; 3 Umeå Centre for Molecular Medicine, Umeå University, 90187 Umeå, Sweden; 4 Global Development, Novo Nordisk A/S, 2860 Søborg, Denmark

**Keywords:** somapacitan, human growth hormone, receptor signaling, P-STAT5 activation, biodistribution, fluorescence molecular tomography, light-sheet fluorescence microscopy, proximal epiphysis

## Abstract

Somapacitan is a long-acting, once-weekly, albumin-binding growth hormone (GH) derivative. The reversible albumin-binding properties leads to prolonged circulation half-life. Here, we investigated and compared somapacitan with human GH on downstream receptor signaling in primary hepatocytes and hepatocellular models and using isothermal titration calorimetry to characterize receptor binding of somapacitan in the presence or absence of human serum albumin (HSA). With non-invasive fluorescence imaging we quantitatively visualize and compare the temporal distribution and examine the tissue-specific growth hormone receptor (GHR) activation at distribution sites. We found that signaling kinetics were slightly more rapid and intense for GH compared with somapacitan. Receptor binding isotherms were characterized by a high and a low affinity interaction site with or without HSA. Using in vivo optical imaging we found prolonged systemically biodistribution of somapacitan compared with GH, which correlated with plasma pharmacokinetics. Ex vivo mouse organ analysis revealed that the temporal fluorescent intensity in livers dosed with somapacitan was significantly increased compared with GH-dosed livers and correlated with the degree of downstream GHR activation. Finally, we show that fluorescent-labeled analogs distributed to the hypertrophic zone in the epiphysis of proximal tibia of hypophysectomized rats and that somapacitan and GH activate the GHR signaling in epiphyseal tissues.

## 1. Introduction

Children and adolescents with growth hormone deficiency have been treated with recombinant human growth hormone (GH) for more than 30 years with positive clinical outcomes [1]. The current treatment regimen is daily subcutaneous injections, which can be a burden for the patients (and parents) and affect the overall treatment adherence and thus, ultimately patient treatment outcomes [2,3,4,5].

Somapacitan is a long-acting, non-covalent, albumin-binding GH derivative for once-weekly dosing. The albumin-binding fatty-acid moiety is covalently attached to GH via a specific linker attached to a single amino acid in the GH backbone [6]. The prolonged circulation half-life of somapacitan is attributed by the reversible albumin-binding properties of the molecule [6,7]. More than 99% of the somapacitan molecules are bound to albumin in the circulation and tissue. The remaining free fraction is cleared renally by glomerular filtration and subsequently taken up by the proximal tubular cells via endocytosis or unspecific protein reuptake receptors and degraded in the tubular cells or degraded during the receptor-mediated internalization by the GHR. Preliminary metabolism studies in rats and humans suggest that the albumin-binding moiety and linker sidechain is cleaved as an intact complex from the GH part of the somapacitan molecule before it is extensively degraded.

Under normal physiological conditions, GH is secreted from the anterior pituitary lobe into the circulation and transported primarily to the liver where it binds the GHR [8]. Ligand binding to the transmembrane homodimeric receptor complex induces a conformational change and concomitant phosphorylation of the tyrosine kinase, Janus kinase 2 (JAK2), which in turn lead to activation of downstream signal transducer and activator of transcription 1 (STAT1), STAT3 and STAT5 [8,9]. Ligand binding and GHR activation also results in direct activation of Src family kinases (SFKs), independent of JAK2, and leads to activation of mitogen-activated protein (MAP) kinase. The STAT transcriptional regulators dimerize and bind to GH-responsive elements in promoter regions of target genes and activate transcription. STAT5 is the major mediator and predominant transcription factor that mediates GH-induced cell proliferation through the upregulation of IGF-1 [8,9].

In children and adolescents, GH is the major regulator of longitudinal growth, acting both via liver-dependent upregulation of insulin-like growth factor I (IGF-1) and via direct actions of GH on the GHR in peripheral tissues. This leads to both autocrine and paracrine IGF-1 effects in bone and muscle tissue. Also, GH affects adipose tissue, ovarian preantral follicle development, increase muscle fiber size by fusion of myoblasts with nascent myotubes, activates neural stem cells, and stimulate chondrocytes proliferation by more direct and less IGF-1 dependent actions [10]. GH effect on adipose tissue has direct clinical bearing for adult patients with growth hormone deficiency, where a reduction in body fat mass and in particular in visceral fat mass is an essential part of the treatment strategy. GH effects on adipose tissue has been reviewed by Kopchick et al [11].

The molecular mechanisms for GHR activation and signaling have been extensively studied in cellular models with exogenously over-expression of the GHR [9] and have provided a good understanding of the plethora of roles played by GH in physiology and disease [8,9]. GH deficiency has been studied postnatally in the surgical hypophysectomy model, and in several gene knockout (KO) and mutant GHR models, which have provided extensive insight to the role of GH in postnatal growth and development [12,13].

*Igf1* KO mice have significant compromised growth, measured by both reduced body length and mass compared with naïve mice. Moreover, the *igf1* KO mice have an observed high incidence of neonatal lethality [12]. Hepatic specific *igf1* KO mice had significantly reduced levels of circulating IGF-1 (more than 80%), but strikingly only a minor reduction in body length and mass [14,15]. Together, the findings suggest that although most circulating IGF-1 is produced in the liver in response the GH stimulation, it is not the amount of IGF-1 that drives the postnatal growth and development alone. Biodistribution of biologics can be studied live in vivo using fluorescence molecular tomography (FMT) [16]. This is a quantitative and high sensitivity optical imaging modality for non-invasive imaging in small animals. FMT provides real-time deep-tissue imaging of biological processes and concomitantly highly sensitive quantification, which enable temporal and organ specific characterization in vivo [17]. The near infrared (NIR) wavelengths provide the spectral window for in vivo imaging as this is the region of least light scattering, low attenuation of light by tissue absorption and decreased level of autofluorescence > 600 nm [18].

In this study, we compared downstream receptor signaling in cellular models of somapacitan and human GH. We used longitudinal non-invasive fluorescence imaging in small animal models to quantitatively visualize and compare the temporal in vivo distribution of the two compounds and to examine the tissue specific GHR activation at the sites of distribution. To substantiate these findings, we analyzed the GHR activation in target tissues.

## 2. Results

### 2.1. Kinetics of Dose and Time-Dependent GH Receptor Activation

Primary rat hepatocytes and human hepatoma cells, HuH-7, were used to study the dose and time-dependent kinetics of direct GHR activation by GH and somapacitan. The level of activation was measured by down-stream tyrosine phosphorylation of STAT5, the primary mediator of IGF-1 transcription. Importantly, both the primary rat hepatocytes and the HuH-7 cells express endogenous levels of GHR, and here we show, for the first time, that these cells can be used for studying endogenous GHR signaling.

**Figure 1:**
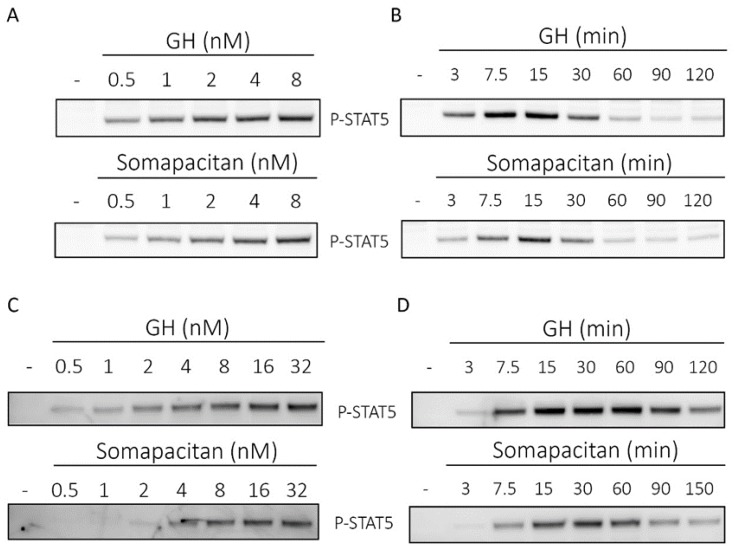
Direct comparison of growth hormone and somapacitan signaling kinetics. Primary rat hepatocytes (**A**,**B**) and human hepatoma cells (**C**,**D**) were treated with either GH or somapacitan. Concentration (**A**,**C**) or time-dependent (**B**,**D**) down-stream receptor activation was analyzed using SDS-PAGE and Western blotting with phosphorylated STAT5 (P-STAT5) antibody. (**A**) Primary rat hepatocytes were treated with a concentration gradient ranging from 0.5 to 8 nM of either GH or somapacitan for 15 min. (**B**) Primary rat hepatocytes were treated with 8 nM GH or somapacitan for a time range of 3 to 120 min. (**C**) Human hepatoma cells were treated with a concentration gradient ranging from 0.5 to 32 nM of either GH or somapacitan for 15 min. (**D**) Human hepatoma cells were treated with 8 nM of either GH or somapacitan and ligand-stimulation terminated at different time points ranging from 3 to 120 min. Equal amounts of protein loaded. Blots shown are one representative of three repeated experiments.

In primary rat hepatocytes, we observed a dose (Figure 1A) and time-dependent (Figure 1B) activation of phosphorylated STAT5 (P-STAT5) for both GH and somapacitan. The kinetics of GHR signal transduction were marginally more rapid and intense for GH compared with somapacitan. We observed clear dose-dependent activation of P-STAT5 from 0.5 nM to 8 nM after 15 min of stimulation (Figure 1A). For both somapacitan and GH, when using the maximum concentration (8 nM) of ligands we observed that maximum P-STAT5 activation was reached between 15 and 30 min post ligand challenge (Figure 1B). Hereafter, comparable ligand-induced GHR desensitization is observed as evident by decreased levels of P-STAT5. Semi-quantification of western blots of P-STAT5 is shown in Supplementary Figure S1.

Next, to examine GHR signal transduction in a human cell system, we used the human liver cells, HuH-7, to compare the time and dose-dependent activation of P-STAT5 after GH or somapacitan treatment. We observed a clear dose-dependent activation of P-STAT5 in response to both somapacitan and GH (Figure 1C) after 15 min of stimulation. When comparing the activation of P-STAT5 by somapacitan with GH, we found that in the low concentration ranges (0.5–2 nM), a higher p-STAT5 response was mediated by GH compared with somapacitan. At the higher concentration range (8–32 nM) both somapacitan and GH showed similar levels of P-STAT5 activation (Figure 1C). Furthermore, these experiments were repeated under serum-starved conditions and exogenous addition of physiological levels of albumin together with a dilution range of the ligands. No difference in the P-STAT5 activation was observed when comparing the presence and absence of albumin.

Furthermore, in HuH-7 cells, time-dependent stimulation studies were carried out with 8 nM of either ligand. We observed a slight delay in the P-STAT5 response in somapacitan-treated cells compared with those treated with GH (Figure 1D). For GH-treated cells, P-STAT5 was already detected 3 min post stimulation and maximal phosphorylation intensity observed between 15 and 30 min post stimulation (Figure 1D). In cells treated with somapacitan, initial P-STAT5 activation was detected at 7.5 min post-stimulation and peak phosphorylation observed between 15 and 30 min after ligand addition (Figure 1D). Hereafter, GHR desensitization was evident with both ligands.

Thus, in primary rat hepatocytes and the human liver cell line, we observed dose and time-dependent activation of GHR signaling measured by tyrosine phosphorylation of STAT5 for both somapacitan and GH. The kinetics of signaling were marginally more rapid and intense for GH compared with somapacitan whereas the desensitization phase for the receptor was similar for the two compounds.

### 2.2. Thermodynamic Parameters of Somapacitan-Receptor Interaction

**Figure 2:**
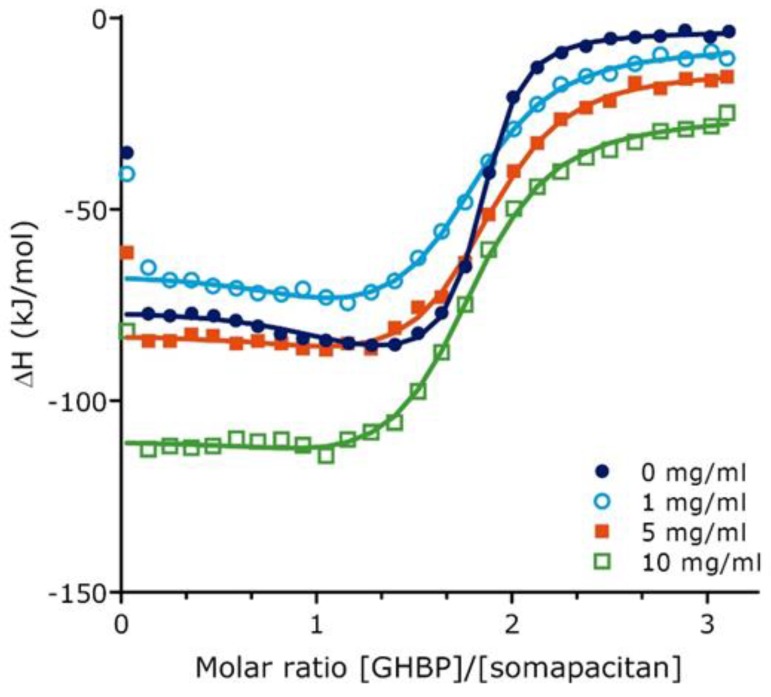
Growth hormone binding protein binding to somapacitan and effect of human serum albumin as measured by isothermal titration calorimetry. Integrated binding isotherms for GHBP (155 µM) binding to Somapacitan (10.2 µM) in absence of HSA (0 mg/mL HSA) (depicted in dark blue) and in presence of increasing concentrations HSA (1mg/mL in light blue, open circle; 5 mg/mL orange, square; 10 mg/mL green; open square). Data treatment was done using a two sets of site fitting model with a fitted offset compensating for the heat of dilution. Binding parameters are reported as the average of individual runs.

To further characterize the binding of somapacitan to its receptor, we employed isothermal titration calorimetry (ITC) in the presence or absence of human serum albumin (HSA). This technique directly measures the heat of interaction between two binding partners in solution, which can be resolved to characterize the binding stoichiometry (N), disassociation constant (KD), change in enthalpy (ΔH), and change in entropy (ΔS). For these studies we used the high affinity growth hormone binding protein (GHBP), which represent the cleaved extracellular ligand-binding domain of the GHR. Binding experiments were performed in the presence of different HSA concentrations (0, 1, 5 and 10 mg/mL). Individual isothermals and integrated binding isotherms at different HSA concentrations are shown in Supplementary Figure S2. In absence of HSA, the binding isotherms were well characterized by a two-site fitting model resulting in a high affinity interaction site (N1 = 0.97, KD1 = 9.1 nM, ΔH1 = −73.9 kJ/mol, −TΔS1 = 26.1 kJ/mol) and a low affinity interaction site (N2 = 0.88, KD2 = 103.9 nM, ΔH2 = −88.1 kJ/mol, −TΔS2 = 46.6 kJ/mol) (Figure 2, Supplementary Table S1 and Supplementary Table S2). Both binding sites exhibited exothermic binding heats. As observed in Figure 2, at HSA concentrations of 0, 1 and 5 mg/mL, the difference between ΔH1 and ΔH2 is more than 8 kJ/mol. Therefore, we were able to resolve thermodynamic binding parameters for the two sites. At higher HSA concentrations of 10 mg/mL, the difference between ΔH1 and ΔH2 is less than 8 kJ/mol and, therefore, the binding response from the high affinity site is not very distinguished from the binding response from the low affinity site. As a result, at 10 mg/mL HSA, only the site 2 can be well characterized by fixing the KD1 to 65 nM. Characterization of binding isotherms indicate that both disassociation constants, KD1 and KD2, are increased approximately five-fold in presence of 1 mg/mL HSA. Further increase in HSA concentration to 5 mg/mL does not lead to a further increase in both disassociation constants.

### 2.3. Fluorescent in Vivo Optical Imaging Describes Clear Temporal Biodistribution Differences of GH and Somapacitan in Mice

**Figure 3:**
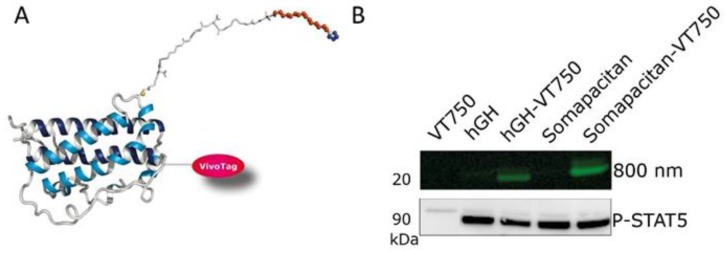
Qualification of fluorescently labelled GH and somapacitan. (**A**) GH analogs were random labelled with the near-infrared fluorescent label VT-750, here is shown somapacitan randomly labelled with VT-750. (**B**) Labelled analogs were qualified in primary rat hepatocyte stimulation assay at 8 nM for 15 min treatment. Cell lysates were run on SDS-PAGE, gel scanned at 800 nm to confirm labelling, and Western blotted with P-STAT5. Included as controls were unlabeled GH and somapacitan and VT750 tag alone.

**Figure 4:**
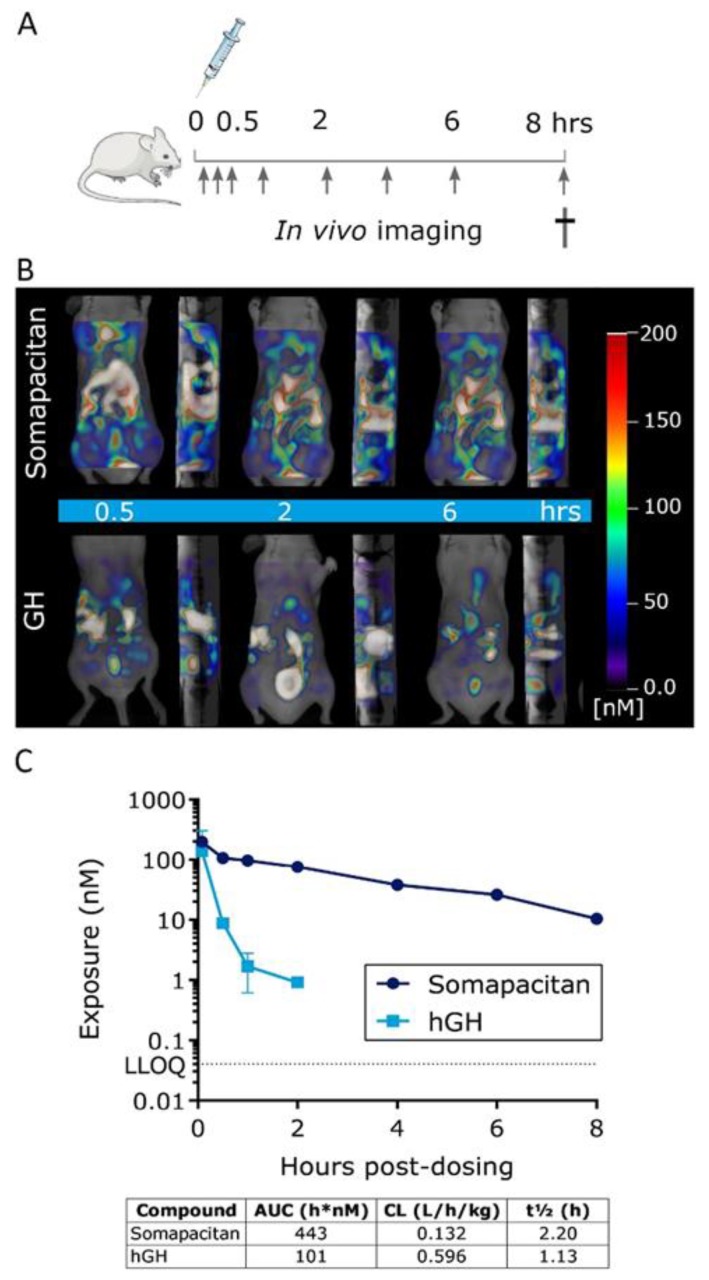
GH and somapacitan show district temporal patterns of biodistribution in mice. (**A**) Schematic illustration of the in vivo study design, arrows indicate fluorescent imaging time points post-dosing. (**B**) Representative frontal and sagittal images of the same animals showing the longitudinal biodistribution of somapacitan and GH at 0.5, 2, and 6 h post iv administration of 60 nmol/kg. Scale bars of fluorescent intensities are included for reference with the highest signal given in white-reddish (200 nM) and lowest intensity depicted in blue (~50 nM). (**C**) Plasma exposure analysis of somapacitan and GH analogs presented on a logarithmic scale. LLOQ: Lower limit of quantification.

Having compared the binding kinetics of the somapacitan and GH to the GHR, we next compared and characterized the spatio-temporal biodistribution of somapacitan and GH in vivo. For this purpose, proteins were labelled with the NIR fluorescent tag; VivoTag^®^750-S (VT750-S), with a single fluorophore per protein (Figure 3A). Previous experiments had shown that randomly labelled GH proteins with high fluorophore:protein ratios gave inconsistent and poorly reproducible results. As a result, it was decided to prepare labelled somapacitan and GH with higher homogeneity. Taking advantage of the highly negatively charged VT750-S, mono-labelled proteins were isolated from random lysine-based labelling mixtures using anion exchange chromatography. More than one mono-labelled species was present in both preparations, but the exact sites of conjugation were not determined. Fluorescently tagged somapacitan and GH are hereafter referred to as somapacitan-VT750 and GH-VT750, control free VT750. The bright and stable ligands were confirmed to retain the pre-labelling GHR activation potential in primary rat hepatocytes (Figure 3B). This was measured by stimulating cells with 8 nM ligands for 15 min and comparing the level of P-STAT5 activation for both ligands. Parental unlabeled proteins and free fluorescent dye VT750 were used as controls. The NIR-labelled GH and somapacitan retained their receptor binding properties and no difference in P-STAT5 activation was observed compared to parental ligand controls. Next, the longitudinal biodistribution pattern was described in spontaneous nude, SKH-1, mice using FMT. The labelled analogs were injected via the tail vein at a concentration of 60 nmol/kg. Mice were subsequently anaesthetized and in vivo imaged with FMT at 0.5, 1, 2, 4, and 6 and 8 h post-dosing (Figure 4A,B). Micro plasma samples were collected throughout the course of the experiment in a sparse sampling regimen. After administration, somapacitan-VT750 was observed throughout the experiment; in the heart and circulation and in highly vascularized organs such as the liver (Figure 4B top panel). GH-VT750, on the contrary, was rapidly cleared from the circulation and a high fluorescent signal was observed immediately after dosing in the kidneys and bladder, indicative of fast renal clearance. The kidney–bladder signal persisted up to 6 h. post-dosing at decreasing intensities (Figure 4B lower panel). These findings were supported by a non-compartmental PK analysis of the plasma exposure data, which revealed, a 4.5-fold reduction in clearance when somapacitan is compared with hGH, clearly consistent with the plasma profile for somapacitan-VT750 compared with GH-VT750 in the SKH-1 mice (Figure 4C).

**Figure 5:**
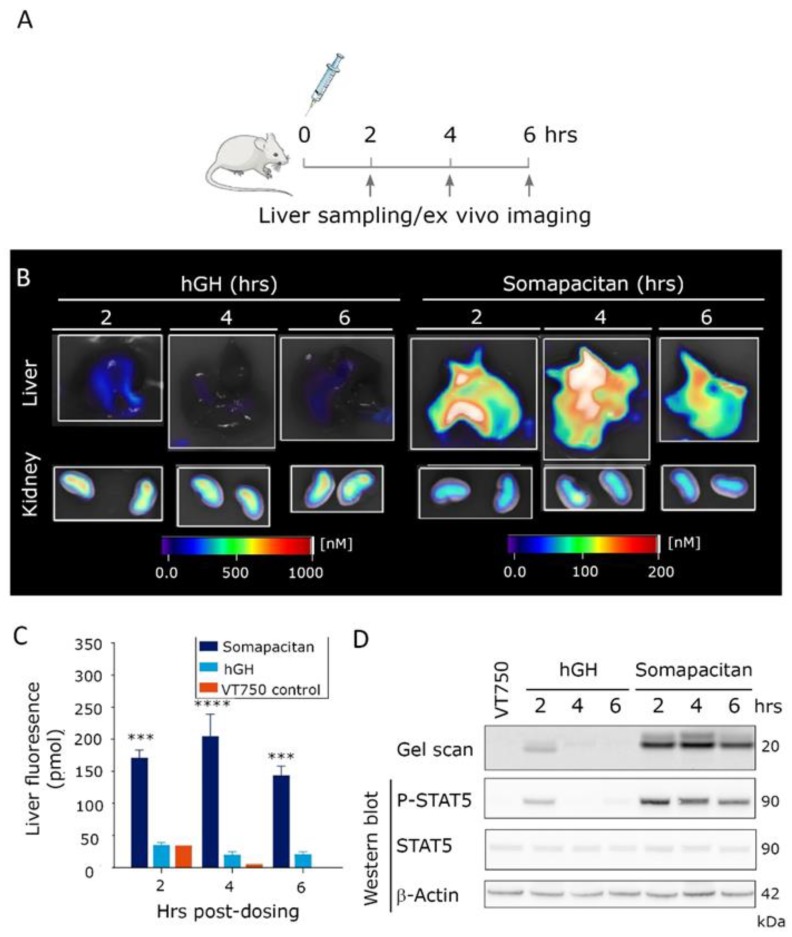
Prolonged residence time and activity of somapacitan in target organs. (**A**) Schematic illustration of ex vivo imaging study design, arrows indicate times for organ harvesting. (**B**) Livers and kidneys of mice dosed GH or somapacitan harvested at 2, 4 and 6 h post-dosing. Fluorescent imaging scale bars are included for reference with scale bars for livers on the right and for kidneys on the left. Highest signals of fluorescence intensities are given in white-reddish (1000 nM for livers and 200 nM for kidneys), lowest intensity depicted in blue (0 nM). (**C**) Temporal quantification of total liver fluorescent intensity (pmol). Somapacitan is shown in dark blue, GH in light blue and VT750 control in orange. Data are shown as mean ± SEM, **** *p* ≤ 0.0001, *** *p* ≤ 0.001 depict somapacitan compared with GH (*n* = 3 livers per condition). (**D**) Homogenized organs harvested at 2, 4 and 6 h post-dosing, separated on SDS-PAGE. Top lane gel scan acquired at 800 nm, protein size of 20 kDa indicated. Lower bands: Corresponding Western blot of P-STAT5, STAT5 and β-actin loading control. Protein sizes in of 90 and 42 kDa are indicated.

Next, to examine the level of GHR activation in major organs of distribution, the organs were harvested 2, 4 and 6 h post-iv dosing of 60 nmol/kg somapacitan-VT750, GH-VT750, or free fluorophore control (VT750). The study design is illustrated in Figure 5A. The relative ex vivo fluorescent signal in livers and kidneys, imaged with 2D planar fluorescence in FMT, is shown in Figure 5B. The fluorescent intensity in livers from mice dosed with somapacitan-VT750 was significantly increased compared with mice dosed GH-VT750 (Figure 5B,C). The majority of GH-VT750 signal was observed in the kidneys (Figure 5B). To confirm that this fluorescent signal was indeed from intact and active protein, livers were homogenized and subjected to gel electrophoresis by sodium dodecyl sulfate–polyacrylamide gel electrophoresis (SDS-PAGE). Fluorescent gel scanning at 800 nm confirmed the presence of intact somapacitan-VT750 in livers at all time points measured. GH-VT750 was only present 2 h. post iv. dosing (Figure 5C). Thus, these findings confirm our in vivo imaging data and demonstrate the prolonged half-life somapacitan compared with GH. Furthermore, immunoblotting with an antibody to P-STAT5 confirmed downstream GHR activation in livers from mice dosed with somapacitan at all time points. GHR activation of P-STAT5 GH was only evident 2 h post-dosing (Figure 5C).

### 2.4. Somapacitan and GH Distributes to and Directly Activates P-STAT5 in Epiphyseal Growth Zones

**Figure 6:**
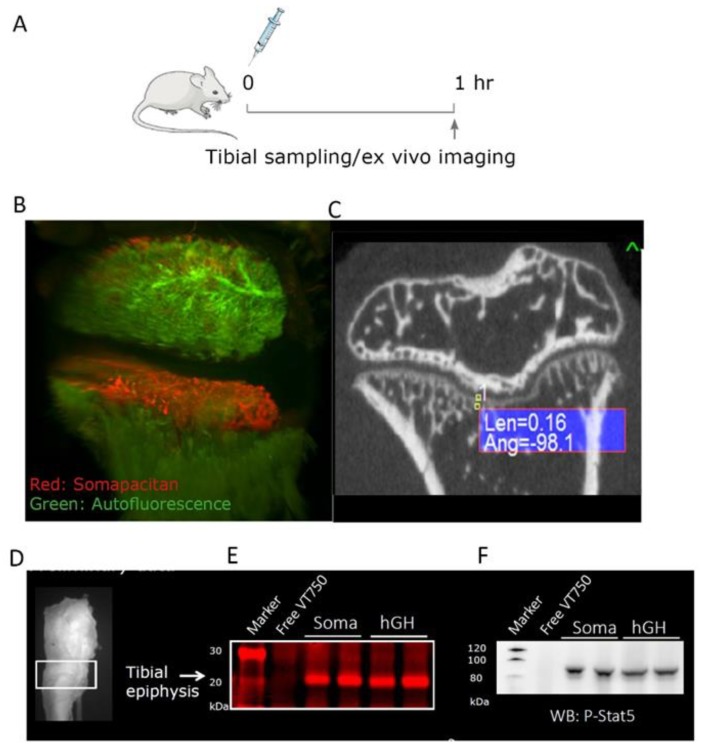
GH analogs can directly activate cells in the proximal epiphyseal tibiae in GH-deficient hypophysectomized rats. (**A**) Illustration of the study design in GH-deficient hypophysectomized rats. Arrow indicate time of ex vivo imaging and termination. (**B**) Ex vivo imaging of tibia from proximal diaphysis to proximal epiphysis of a representative rat 1 h after dosing somapacitan-VT750 in red. Tissue autofluorescence is depicted in green. Bone were scanned with light-sheet fluorescence microscopy, scale bar represents 300 μm. (**C**) Representative bone scan obtained with micro-computed tomography showing the coronal plane of a hypophysectomized rat calcified bone structures for anatomical reference to LSFM 3D reconstructions. (**D**) Illustration of the dissected part of the proximal tibia. (**E**) Homogenized growth zone of the proximal tibiae lysed and separated on SDS-PAGE, gel scanned at 800 nm to detect NIR labelled GH and somapacitan analogs in homogenates. Samples from two individual rats are depicted along with a control dosed uncoupled VT750. (**F**) Corresponding Western blots showing direct activation of the GHR by both somapacitan and GH represented by P-STAT5 activation in the growth zone of GH-deficient rats.

Having compared the biodistribution and direct kinetics of GHR activation in the liver, we next wanted to address and compare the IGF-1-independent signaling in peripheral tissues for somapacitan and GH. We made use of the GH-deficient, post-natal hypophysectomized rat model dosed either of the labelled GH compounds in a bolus of 60 nmol/kg iv in the tail vein and the right legs harvested after 1 h (Figure 6A). To visualize the ex vivo distribution in the proximal tibial epiphysis, we used whole organ light-sheet fluorescence microscopy (LSFM) and for anatomical reference the specimens were scanned with micro-computed tomography (CT). The LSFM imaging system provides high sensitivity, micrometer resolution, and allows for 3D reconstructed image analysis of the organ of interest. We found that, somapacitan-VT750 specifically to the hypertrophic zone and primary spongiosa in the epiphysis of proximal tibia as evident by bright red staining in this region. Auto-fluorescence is shown in green (Figure 6B) and movie of the 3D reconstructed fluorescence scan is available in the Supplementary Figure S3. The corresponding micro-CT planar image of the proximal tibiae is shown for anatomical reference (Figure 6B,C). On this micro-CT representation calcified bone is shown in white and soft tissue in dark.

To further examine the level of GHR activation in this bone region, we harvested the tissue corresponding to the growth zone of in the proximal epiphysis. The tissue was homogenized, lysed and separated by SDS-PAGE as described for liver tissue analysis. In these epiphyseal tissue homogenates, we could clearly detect NIR-labelled GH and somapacitan at the correct size of 20 to 22 kDa (Figure 6E). Markedly, we found strong direct activation of GHR signaling with, both somapacitan and GH, in the growth zone homogenates as measured by P-STAT5 activation (Figure 6F).

## 3. Discussion

In this study we have investigated the downstream receptor signaling in primary rat hepatocytes and human hepatoma cells together with the binding affinity for binding to the GHR of the long-acting GH derivative, somapacitan. We have further visualized and compared the temporal in vivo distribution and GHR activation of somapacitan and GH at the sites of distribution by using longitudinal non-invasive fluorescence imaging.

Strong dose-dependent activation of GH signaling in primary rat and human hepatoma cells was observed with human GH and somapacitan. Human GH gave rise to a minimally faster and marginally stronger P-STAT5 response compared to somapacitan. When further examining the time-dependent kinetics of, a fixed dose of somapacitan and human GH, we observed marginally more rapid P-STAT5 kinetics for GH compared with somapacitan. Thus, we observed dose and time-dependent activation of GHR signaling as measured by tyrosine phosphorylation of STAT5 for both GH and somapacitan. The kinetics of signaling were more rapid and intense for GH compared with somapacitan whereas the desensitization phase for the receptor was similar for the two compounds. We hypothesize that these differences in receptor-induced signaling relate to the differences in receptor affinity.

Equilibrium binding studies using ITC showed that binding between somapacitan and GHBP in the absence of albumin could be characterized by two sets of binding sites with a KD1 of 9.1 nM (high affinity binding site) and a KD2 of 103.9 nM (low affinity binding site) corresponding to a 11-fold difference in affinity between the two binding sites. Binding measurements for GH binding to GHBP performed by surface plasmon resonance analysis also suggested two sets of sites, however with a 5.7- and 27-fold higher binding affinity for GH compared with somapacitan at site 1 and site 2 respectively [19,20]. The difference in binding affinity between somapacitan and GH is consistent with the more rapid and intense signaling kinetics observed for GH in the cellular liver assays. However, especially the much lower binding affinity at site 2 of somapacitan is surprising and unexpected and suggest that this is a result of the different binding assays used or the site 2 binding is less important for GHR signaling, since the side chain covalently attached to the GH backbone of the somapacitan molecule could interfere with the binding to site 2 on GHR/GHBP.

In the presence of albumin, the dissociation constants, KD1 and KD2, for somapacitan were almost equally increased (approximately 5-fold) at 1 mg/mL HSA. Further increase in HSA concentration to 5 and 10 mg/mL did not significantly affect the disassociation constants. Suggesting that somapacitan already bound to albumin can bind the GHR although with an approximately 5-fold lower binding affinity. The reduced binding affinity to the GHR will most likely reduce the receptor-mediated internalization of somapacitan and thereby contribute to the prolonged circulation time of the molecule compared with to human GH.

The spatio-temporal bio-distribution of GH and somapacitan was investigated by FMT, in spontaneous nude SKH-1 mice using fluorescently tagged compounds. Somapacitan-VT750 was observed throughout the experiment; in the circulation, heart, liver, and other highly vascularized organs, whereas GH-VT750 was rapidly cleared from the circulation and found in the kidneys and bladder immediately after dosing, indicative of fast renal clearance. The kidney-bladder signal persisted up to 6 h. post-dosing for both somapacitan-VT750 and GH-VT750, although the GH-VT750 was at decreasing intensities. These findings support previous reports that renal clearance is an important elimination route for GH [21,22], but also suggests a more extensive distribution of somapacitan to primarily highly perfused organs. The presence of active protein was confirmed in liver homogenates run on gel electrophoresis and measured with fluorescent gel scanning and western blotting with P-STAT5 antibody. GHR activation of P-STAT5 in livers from mice dosed with somapacitan was demonstrated at all time points post dosing whereas GHR activation of P-STAT5 after GH administration was only evident 2 h post-dosing. These findings confirm our in vivo imaging data and demonstrate the prolonged half-life of somapacitan compared to GH.

In this study somapacitan and GH were dosed intravenously in order to eliminate the impact of the absorption processes on distribution and clearance of the compounds. In both humans and animals, GH has been reported to show absorption limited elimination after subcutaneous administration, resulting in a delayed absorption and prolonged apparent half-life. Somapacitan, however, do not show absorption-limited elimination in humans or animals, but a relatively fast absorption compared to the elimination half-life. This results in higher exposure and subsequent higher IGF-1 concentration-time profiles after subcutaneous dosing of somapacitan compared to GH. Finally, we investigated the somapacitan and GH-induced signaling in peripheral tissue by visualizing the ex vivo distribution to the proximal tibial epiphysis of GH-deficient hypophysectomized rats. Somapacitan-VT750 specifically distributed to the hypertrophic zone and primary spongiosa in the epiphysis of proximal tibia. This was also confirmed by the presence of somapacitan-VT750 and GH-VT750 in epiphyseal tissue homogenates using SDS-PAGE. In the same epiphyseal tissue homogenate, a strong direct activation of GHR was measured at P-STAT5 level suggesting that somapacitan as well as GH can distribute to the peripheral tibia growth plate and directly stimulating longitudinal growth by activating the GHR.

In conclusion, here we extensively characterize the long-acting, albumin-binding GH derivative somapacitan. We show that somapacitan efficiently activate downstream GHR signaling in primary cells and cells of human liver origin. Furthermore, we describe the binding kinetics to the GHR in the presence or absence of albumin. Strikingly, we show that both somapacitan and human GH can induce STAT5 phosphorylation in target organs such as the liver and mediate direct actions on the GHR in peripheral tissues in growth hormone deficient animals.

## 4. Materials and Methods

### 4.1. Animal Studies

Spontaneous nude female SKH-1 mice or hypophysectomized male Sprague Dawley rats were purchased from Charles River, Sulfeld, Germany. Mice were 4 to 6 weeks old and rats 6 weeks old upon arrival. Rats were hypophysectomized at the vendors facility at 4 weeks of age according to standard procedures. Mice were allowed 1 week of acclimatization prior to the start of the experiment. Animals were allowed free access to water and non-fluorescent rodent chow (Altromine C1039, Altromin, Germany). Rats were used at the age of 14 weeks. Animals were grouped-housed in type IV cages, with ten mice per cage, or five rats per cage with stainless steel mesh lids and bedding of aspen wood shavings. Cages were supplemented with environmental enrichments. Animals were kept on a 12 h light/dark cycle at a constant temperature of 20 to 22 °C. All invasive and live imaging procedures were performed under isoflurane inhalation anesthesia (1–5 vol%, N_2_O 0.7 L/min, O_2_ 0.3 L/min). Animal experiments were conducted in accordance with the EU Directive 2010/63/EU for animal experiments, the Protection of Animals Act, the Act on Experiments on Animals and the Standard Operating Procedures for Experiments on Animals at Novo Nordisk A/S. The experiments were performed under the supervision and approval of the Danish Government Animal Experiments Inspectorate, and the Novo Nordisk Ethical Review Committee.

### 4.2. Micro Plasma Sampling and Analysis

At each sampling time, 1 or 2 droplets of blood (approximately 10 µL) was sampled from the tail by use of a lancet and a 10 µL capillary glass tube coated with Heparin and sealed in one end with wax. The blood sample was kept at room temperature for a maximum of 10 min until centrifugation at 2000×*g* for 5 min at 20 °C. The capillary glass was cut at 13 mm and placed in an Eppendorf tube containing assay buffer. The tube was vortexed until homogeneous in color. Forty microliters was transferred to microtiter plates and stored at −80 °C until analyzed. Plasma concentrations of GH analogs were determined by beads-based luminescence oxygen channeling immuno-assays as previously described [6,23]. The dynamic range for GH was 0.6 to 1000 ng/mL and when a reading was below the lower limit of quantification, the values were set to half of the lower limit, in the case of GH to 0.3 ng/mL.

### 4.3. Cell Culture and Reagents

Primary rat hepatocytes were isolated using in vivo liver perfusion with collagenase in Sprague Dawley rats according to standard techniques [24]. Washed cells were plated at a density of 1.65 × 106 cells/mL on six well plates, pre-coated with collagen type I, at 7.81 µg collagen/cm^2^ (Sigma, Søborg, Denmark) in Williams E medium with GlutaMax, 4 µg/mL insulin, 10% FBS, 100 U/mL penicillin, 100 µg/mL streptomycin. The human hepatoma cell line, HuH-7, were plated on fibronectin coated six well plates at a density of 3.25 × 105 cells/well in DMEM low glucose 5.55 mM and pyruvate (ThermoFischer, Roskilde, Denmark) supplemented with 10% FBS (ThermoFischer, Roskilde, Denmark) and 100 U/mL penicillin, 100 µg/mL streptomycin (ThermoFischer, Roskilde, Denmark).

GH analogs and controls were pre-diluted in culture medium and 10 µL of ligand added per well. Cells were stimulated with a dilution range of ligand concentrations for 15 min For time-dependent studies, cells were stimulated with 8 nM GH-analog for in a time range. After stimulation, cells were washed with ice-cold PBS (ThermoFischer, Roskilde, Denmark) and lysed with RIPA lysis buffer with protease inhibitor cocktail 2 (Roche, Hvidovre, Denmark) and phosphatase inhibitor cocktails (Sigma, Søborg, Denmark). Plates left on ice for 20 min and lysates were collected by scraping and centrifuged at 15,000×*g* for 15 min at 4 °C. Supernatant were stored at −80 °C. Protein amounts were determined using standard techniques and equal amount of protein was loaded onto the gels.

### 4.4. Tissue Homogenization

Snap frozen tibial tissue was transferred to a zip-bag and homogenized using a rubber hammer. Tissue pieces were then transferred to 2 mL Eppendorf tubes and kept on dry ice. Liver samples (300 mg) were cut into small pieces with a scalpel on dry ice. Tissue homogenates were lysed with 1 mL T-PER, tissue protein extraction reagent (ThermoFischer, Roskilde, Denmark) with added protease inhibitors (halt protease inhibitor cocktail, EDTA-Free, (ThermoFischer, Roskilde, Denmark). Liver samples were homogenized with 5 mm stainless steel beads (Qiagen, Aarhus, Denmark) on a Tissue-lyzer II (Qiagen, Aarhus, Denmark). 0.75 mL T-PER reagent was added per tibial sample and bone samples were homogenized using a Tissue Ruptor (Qiagen, Aarhus, Denmark) at 30 Hz for three rounds of 1 min. The samples were then centrifuged at 10,000×*g* for 15 min at 4 °C to pellet cell/tissue debris. The supernatants were collected and kept at −80 °C.

### 4.5. SDS-PAGE, Gel Scanning and Western Blotting

Cell homogenates were separated by SDS-PAGE on NU-PAGE 4% to 12% bis-tris gels in MES running buffer (Invitrogen, 2630 Taastrup, Denmark) according to standard techniques. Gels containing lysates with fluorescently tagged protein were scanned at 800 nm on an Odyssey^®^ gel scanner (Li-Cor Biothechnology, Lincoln, NE, USA) and processed for Western blotting using standard techniques. Antibodies: Anti-P-STAT5 (C11C5 cat. no 9359, Cell Signaling Technology, Danvers, MA, USA) and anti-STAT5 (D206Y cat. no. 94205, Cell Signaling Technology, Danvers, MA, USA) HRP-labelled secondary antibodies.

### 4.6. ITC

Somapacitan and growth hormone binding protein were dialyzed using Slide-A-Lyzer™ dialysis cassettes overnight while HSA was passed through size exclusion chromatography to buffer exchange in 4.38 mM histidine, 2.05 mM NaCl pH 7.1 buffer. All proteins were subjected to SE-HPLC-MALS analysis to determine the concentration before and after the up-concentration. SE-HPLC-MALS was carried out on an Agilent 1200 Series HPLC system (Agilent, Darmstadt, Germany) using a TSK G3000 SWXL column (Tosoh Bioscience, Tokyo, Japan). The column was maintained at a temperature of 20 °C and eluted with 200 mM phosphate (pH 6.8) containing 300 mM NaCl and 4% 2-propanol at a flow rate of 0.8 mL/min. Detection was done by UV absorption at 280 nm, refractive index (RI) and multi-angle laser light scattering (MALS). RI and MALS detectors were Optilab T-rEX (Wyatt, CA, USA) and MiniDawn Treos (Wyatt, CA, USA), respectively.

All ITC experiments were conducted using a PEAQ-ITC calorimeter (Malvern, UK) at 37 °C. The sample cell (200 μL) contained 10.2 µM Somapacitan with differing HSA concentrations (of 0, 1, 5, 10 and 30 mg/mL). GHBP (ligand), at 155 µM, was injected via the syringe (40 μL). A thermal equilibration step was followed by a 60-s delay and subsequently an initial 0.4-μL injection of GHBP, followed by 25 injections of 1.5 μL and a final injection of 1.1 μL at an interval of 120 s. The stirring speed was maintained at 750 rpm, and the reference power was kept constant at 29.3 μW. Experiments were performed as at least duplicates and data treatment done using the PEAQ-ITC analysis software (Malvern Instruments, Malvern, WR, UK) using a two sets of site fitting model with a fitted offset compensating for the heat of dilution.

### 4.7. Fluorescent Labeling

Somapacitan and recombinant GH were labelled with a single NIR fluorescent (NIRF) label VT750-S (Perkin Elmer, Skovlunde, Denmark). The labelling of GH (30 mg) was performed in 20 mM HEPES buffer pH 7.5 at a protein concentration of 15 mg/mL, and with 3.0 eq. of VT750-S NHS ester (Perkin Elmer # NEV10124, Perkin Elmer, Skovlunde, Denmark). After reaction for 1 h at room temperature, the reaction mixture was loaded to a HiTrap Q HP column (GE Healthcare, Brøndby, Denmark) equilibrated with 20 mM triethanolamine buffer pH 8.0 and the mono-derivatized fluorescent conjugates were eluted using a gradient of 0 to 600 mM NaCl over 20 column volumes. The elution profile indicated the presence of more than one mono-labelled species, but the product was devoid unreacted GH and higher PEGylated species as proven by RP-UPLC and MS analysis (found 22991.5, calc. 22990.8). Finally, the product was buffer exchanged to SimplexX buffer (7 mM histidine, 39 mg/mL mannitol, 3 mg/mL Poloxamer 188, pH 7.0) on a Sephadex G25-fine column (GE Healthcare, GE Healthcare, Brøndby, Denmark). Yield: 4.0 mg (13%). Using a similar protocol, somapacitan (60 mg) was mono-labelled using 2.7 eq. of VT750-S NHS ester. MS characterization: found 24172.1, calc. 24171.2. Yield 12 mg (20%).

### 4.8. In Vivo and ex Vivo Optical Fluorescent Imaging

The FMT was calibrated according to standard procedures [16] using dilution ranges of labelled GH and somapacitan in droplets of 20 μL. For trans-illumination scanning the FMT instrument settings were adjusted to 3 mm density scans and high intensity with laser settings of excitation at 750 nm and emission at 780 nm. Acquired raw data were reconstructed using TrueQuant software (PerkinElmer, Skovlunde, Denmark) and the fluorescent signals in a given user defined volume of interest (VOI) were measured. The read-out being pmol for 3D reconstructed tomography or counts/s for 2D planar epi-illumination images. Animals were randomized with *n* = 9 per condition. Fluorescently labelled proteins were dosed as a bolus intravenously (iv) via the tail vein at 60 nmol/kg at a dose volume of 5 mL/kg for mice and 3 mL/kg for rats.

The live temporal biodistribution of fluorescently labelled GH analog was followed in the same animal over time and the signal intensification quantified at various time points from 0 to 6 h post-administration. The imaging procedure was performed as follows; the animal was placed in an optical scanning cassette under constant isoflurane anesthesia and the cassette transferred to the pre-heated FMT imaging chamber for transillumination laser scanning. For ex vivo imaging, the organs of interest were collected after in vivo perfusion with sterile lukewarm saline 2, 4 and 6 h. post-dosing. Organs were placed on a non-scattering scanning block, imaged, and quantified.

Hypophysectomized rats were euthanized 1 h post dosing by in vivo perfusion with sterile saline in deep anesthesia, the proximal tibiae were collected, and scanned ex vivo as described for the mice.

### 4.9. Micro-CT

At termination the proximal tibiae of representative rats were scanned using micro-CT with a Quantum FX^®^ (Perkin Elmer, Waltham, MA, USA). Bones were fixated in a custom made styrofoam holder and scanned at: Field of view of 10 mm, 20 µm voxel size, 200 ms integration time, 90 kV and 160 µA. Sagittal 2D representations of the proximal tibia were used for anatomical reference of LSFM scans. The tibiae were next transferred to PFA and processed for whole organ fluorescent imaging.

### 4.10. Ex Vivo LSFM

Tissues were fixated in 4% PFA for 4 h. and then dehydrated in three steps at 12 h each: 50% ethanol at pH = 9; 70% ethanol pH = 9; and lastly, twice in 100% ethanol. The bones were then transferred to ethyl-3-phenylprop-2-enoate. Fluorescence organ imaging was performed with a light sheet ultramicroscope coupled to a SuperK EXTREME (EXR-15) laser system (LaVision, Ypsilanti, MI, USA). The bone samples were mounted into a specific sample holder, immersed in the sample chamber and scanned in 5 μm steps at excitation/emission settings of 620/700 nm for autofluorescence and sompacitan-VT750 at 710/775 nm. Images and 3D reconstructions were generated using Imaris Bitplane software (Imaris x64 7.5.1, Oxford instruments, Abingdon, UK).

### 4.11. Statistics

Statistical analysis was performed using GraphPad Prism 7 version 7.04, (GraphPad Software, La Jolla, CA, USA). When comparing the mean over time, two-way ANOVA with Sidak’s post-test was used. A *p* value of < 0.05 was accepted as statistically significant.

## Supplementary Materials

Supplementary materials can be found at https://www.mdpi.com/1422-0067/21/4/1181/s1.

## Abbreviations

ΔH	Change in enthalpy
ΔS	Change in entropy
CT	Computed tomography
FMT	Fluorescence molecular tomography
GH	Human growth hormone
GHBP	Growth hormone binding protein
GHR	Growth hormone receptor
HSA	Human serum albumin
IGF-1	Insulin-like growth factor 1
ITC	Isothermal titration calorimetry
JAK2	Janus kinase 2
KD	Dissociation constant
KO	Knockout
LSFM	Light-sheet fluorescence microscopy
N	Binding stoichiometry
NIR	Near infrared
SFKs	Src family kinases
SDS-PAGE	Sodium dodecyl sulfate–polyacrylamide gel electrophoresis
STAT	Signal transducer and activator of transcription
VT750S	VivoTag^®^750-S
