# Supplementary material for: Tissue Distribution and Receptor Activation by Somapacitan, a Long Acting Growth Hormone Derivative
Source: Int J Mol Sci. 2020 Feb 11;21(4):1181. doi: 10.3390/ijms21041181 (PMC7072805; doi:10.3390/ijms21041181)

Supplement for:

Tissue distribution and receptor activation by somapacitan, a long acting growth hormone derivative

Maj Petersen^1^, Prafull S. Gandhi^2^, Jens Buchardt^2^, Tomas Alanentalo^3^, Johannes Josef Fels^2^, Nils Langeland Johansen^2^, Peter Helding-Kvist^1^, Knud Vad^4^, Peter Thygesen^1*^

^1^ Global Drug Discovery, Novo Nordisk A/S, 2760 Måløv, Denmark; [mjpr@novonordisk.com](mailto:mjpr@novonordisk.com) (MP), [phkv@novonordisk.com](mailto:phkv@novonordisk.com) (PHK), [ptt@novonordisk.com](mailto:ptt@novonordisk.com) (PT)

^2^ Global Research Technologies, Novo Nordisk A/S, 2760 Måløv, Denmark; [pgan@novonordisk.com](mailto:e-mail@e-mail.com) (PSG), [jbuc@novonordisk.com](mailto:jbuc@novonordisk.com) (JB), [jjf@novonordisk.com](mailto:jjf@novonordisk.com) (JJF), [nils@hoerup-langeland.dk](mailto:nils@hoerup-langeland.dk) (NLJ)

^3^ Umeå Centre for Molecular Medicine, Umeå University, 90187 Umeå, Sweden; [tomas.alanentalo@umu.se](mailto:tomas.alanentalo@umu.se) (TA)

^4^ Global Development, Novo Nordisk A/S, 2860 Søborg, Denmark; [vad@novonordisk.com](mailto:vad@novonordisk.com) (KV)

***** Correspondence: ptt@novonordisk.com; Tel.: +45-30754617 (PT)

**Table S1 Equilibrium binding parameters for the high affinity binding site at increasing concentrations of HSA using ITC.**

| **HSA** | **High Affinity Site 1** | | | | |
| --- | --- | --- | --- | --- | --- |
|  | **N1** | **KD1** | **ΔH1** | **ΔG1** | **-TΔS1** |
| mg/mL |  | nM | kJ/mol | kJ/mol | kJ/mol |
| 0 | 0.97 | 9.1 | -73.9 | -47.8 | 26.1 |
| 1 | 0.93 | 47.2 | -59.7 | -43.5 | 16.2 |
| 5 | 0.99 | 49.8 | -69.0 | -43.4 | 25.6 |
| 10 | 0.98 | 65.0 (fixed) | -85.3 | -42.7 | 42.6 |

**Table S2 Equilibrium binding parameters for the low affinity binding site at increasing concentrations of HSA using ITC.**

| **HSA** | **Low affinity site 2** | | | | | **Offset** | **Red. Chi Sq** |
| --- | --- | --- | --- | --- | --- | --- | --- |
|  | **N2** | **KD2** | **ΔH2** | **dΔG2** | **-TΔS2** |  |  |
| mg/mL |  | nM | kJ/mol | kJ/mol | kJ/mol | kJ/mol | (kJ/mol)^2^ |
| 0 | 0.88 | 103.9 | -88.1 | -41.5 | 46.6 | -5.1 | 1.3 |
| 1 | 0.89 | 566.7 | -74.0 | -37.1 | 36.9 | -5.7 | 1.0 |
| 5 | 0.91 | 473.0 | -79.0 | -37.6 | 41.4 | -14.2 | 1.3 |
| 10 | 0.78 | 608.0 | -93.5 | -37.0 | 56.6 | -24.3 | 2.3 |

## Supplementary Figure 1. Semi-quantification of western blots of P-STAT5. Primary rat hepatocytes stimulated in *A.* Concentration and *B.* Time-dependent manner with GH (black bars) vs somapacitan (white bars). *C*. Time-dependent P-STAT5 quantification in human liver carcinoma cells. Graphs depict relative light units (AU). Protein loads are normalised prior to loading on the gel.

##
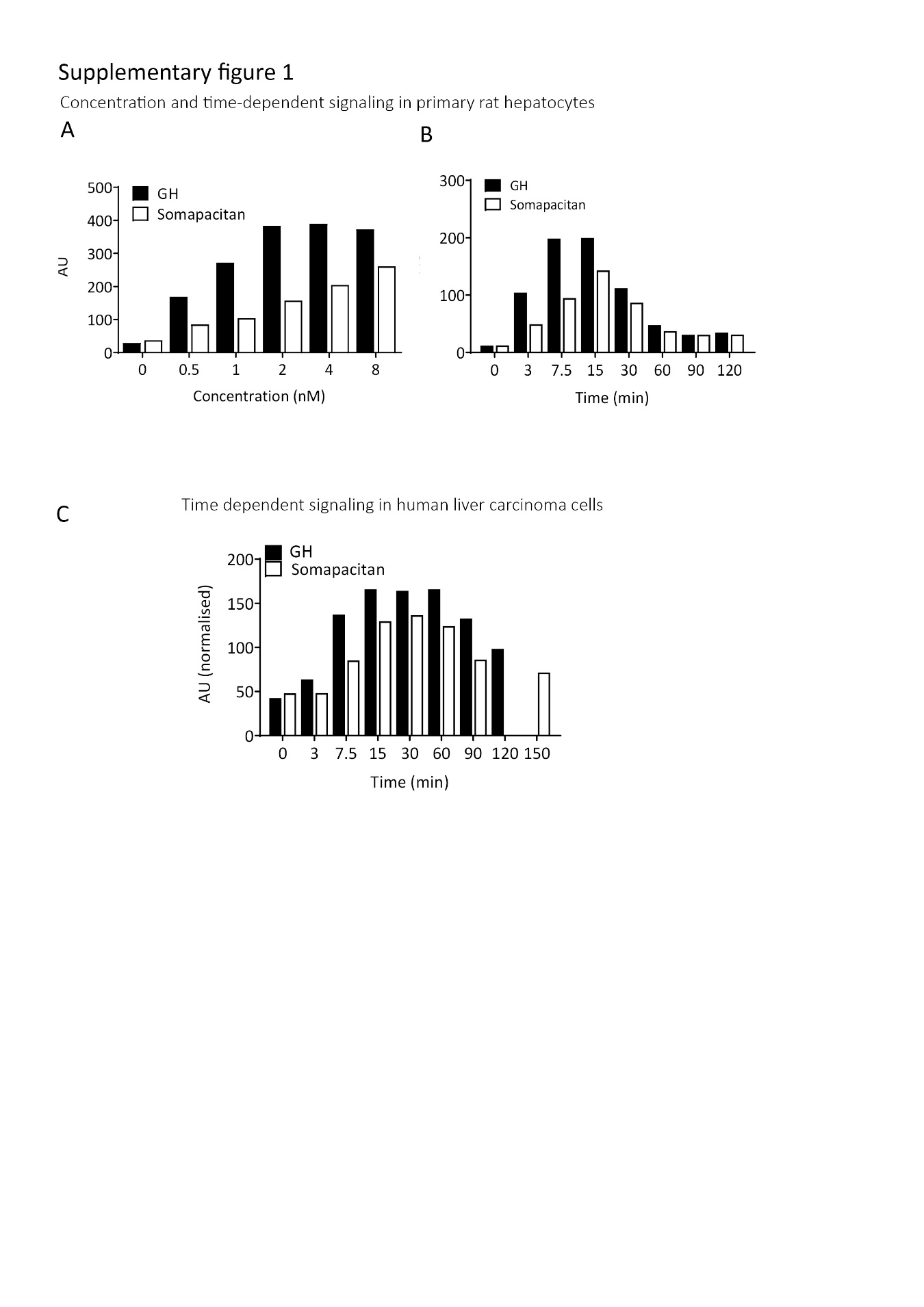


## Supplementary Figure 2. Individual isothermal. Raw heat of interaction (top panel) and Integrated binding isotherm (bottom panel) for GHBP (155 µM) binding to Somapacitan (10.2 µM) in absence of HSA (0 mg/ml HSA) and in presence of increasing concentrations of HSA. Data treatment was done using the PEAQ-ITC analysis software (Malvern Instruments) using a two sets of site fitting model with a fitted offset compensating for the heat of dilution. Binding parameters reported in main text are average of individual runs.

## 0 mg/ml HSA

##
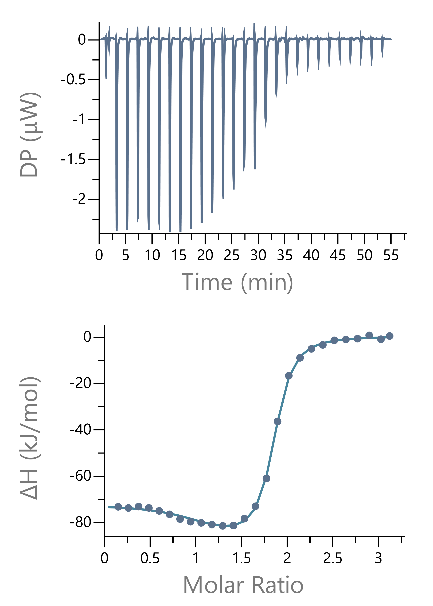

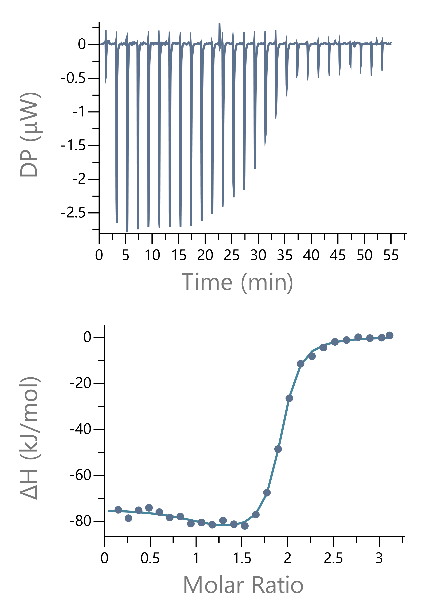


## 1 mg/ml HSA

##
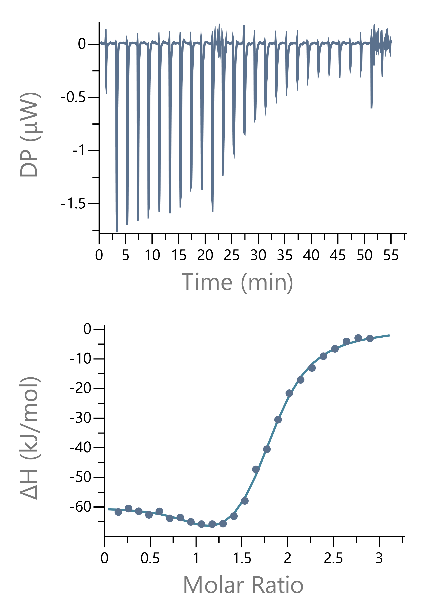

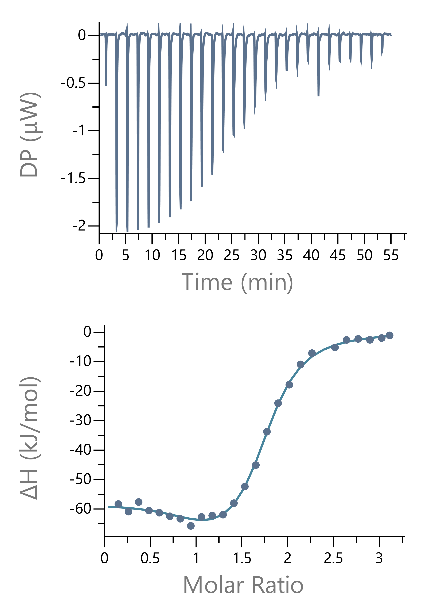

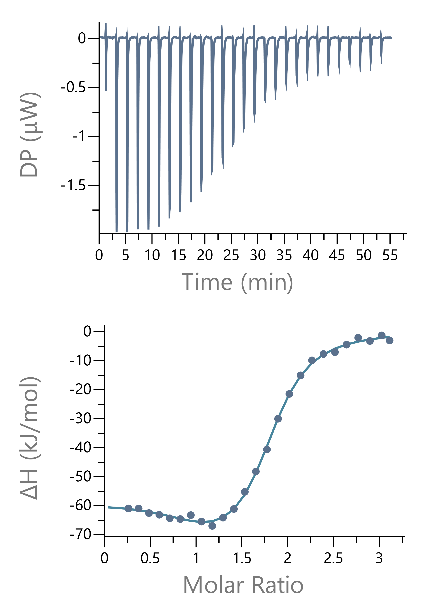


## 5 mg/ml HSA

##
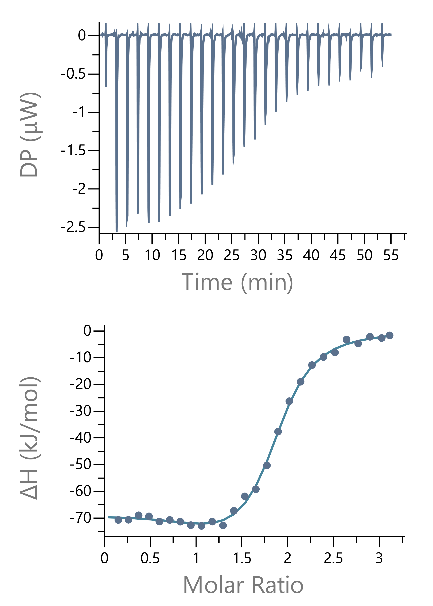

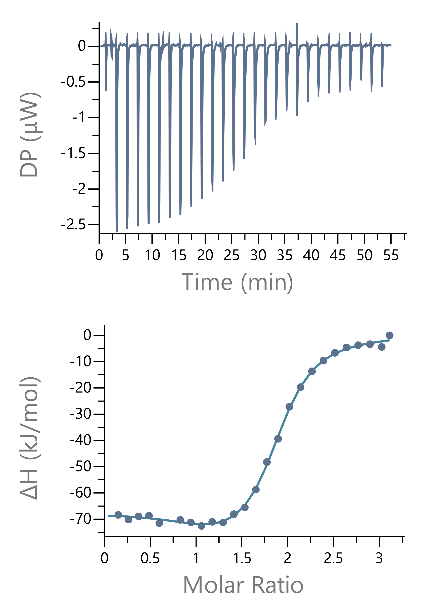


## 10 mg/ml HSA

##
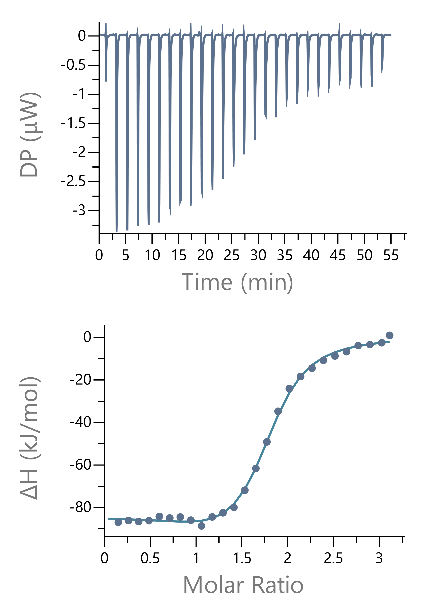

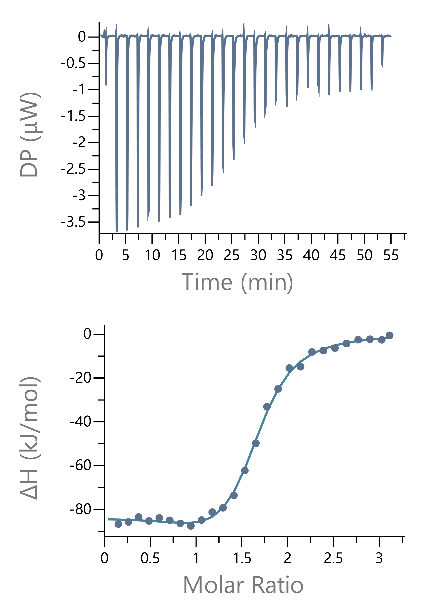


## Supplementary Figure 3. Somapacitan distributes to tibial epiphyseal growth zones in GH-deficient HYP rats. 3D reconstructed representation of LSFM showing somapacitan (in red) and tissue autofluorescence (in green). Somapacitan distribution is observed in the hypertrophic growth zone of the proximal tibial in GH-deficient HYP rats 1 hr after iv dosing.


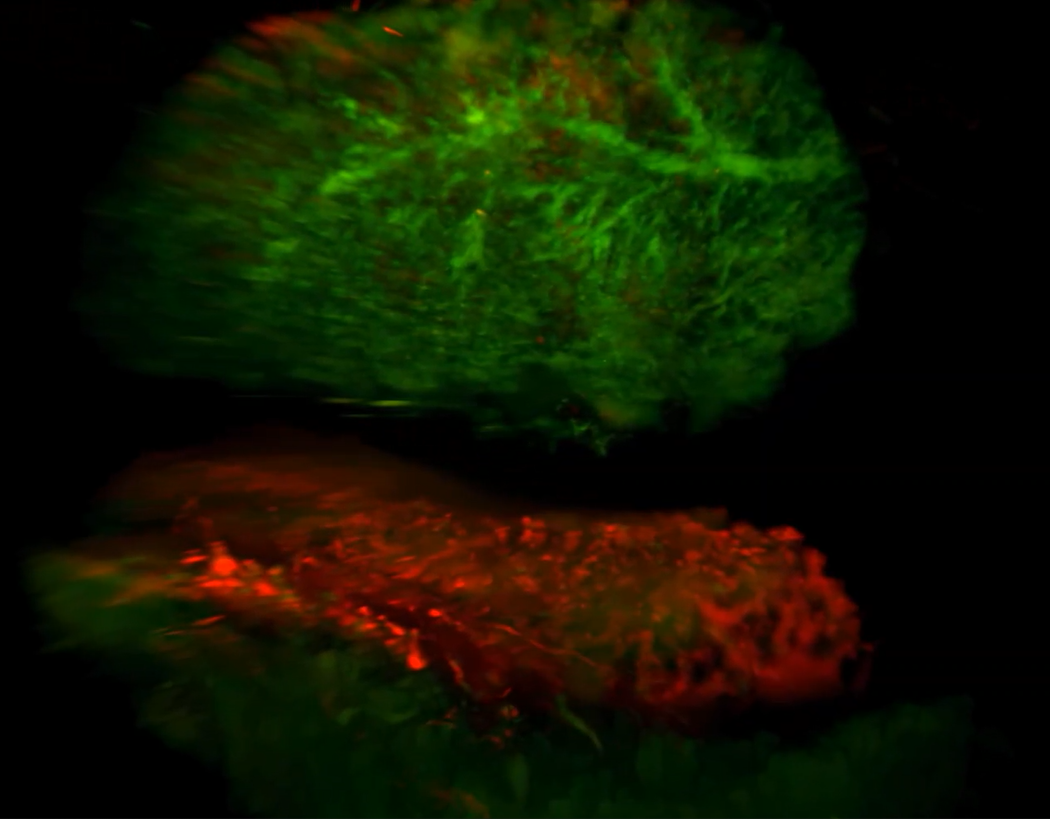

Supplement: Supplementary file 1 [file ijms-21-01181-s001.zip › supplementary update/ijms-710687-supplementary.docx]
